# Breaking the seizure cycle: Belgian expert consensus on the diagnostics and treatment of acute convulsive seizures in children

**DOI:** 10.1007/s13760-026-03045-5

**Published:** 2026-05-22

**Authors:** A. Aeby, B. Ceulemans, K. Jansen, A. Jansen, L. Lagae, P. Leroy, A.-S. Schoonjans, H. Verhelst, M. R. Cilio

**Affiliations:** 1 Present Address: Department of Paediatric Neurology, Hôpital Universitaire des Enfants Reine Fabiola (HUDERF) et Hôpital Erasme, Member of European Reference Network EPICARE, Université Libre de Bruxelles (ULB), Hôpital Universitaire de Bruxelles (H.U.B), Brussels, Belgium; 2 Department of Pediatric Neurology, Antwerp University Hospital (UZA), Edegem, Belgium; 3 Section Pediatric Neurology, Department Development and Regeneration, Member of European Reference Network EPICARE, University Hospitals KU Leuven, Leuven, Belgium; 4 Departement of Pediatric Neurology, CHU de Liège, Liège, Belgium; 5 Department of Paediatrics, Division Paediatric Neurology, Ghent University Hospital, Ghent, Belgium; 6 Division of Child Neurology, Department of Pediatrics, Institute of Neuroscience (IoNS), Cliniques Universitaires Saint-Luc, Université Catholique de Louvain, Brussels, Belgium

**Keywords:** Febrile seizure, Outpatient, Recommendations, Benzodiazepine, Levetiracetam

## Abstract

**Introduction:**

Seizures are the most common neurological emergency in children. Heterogeneous causes, subtypes, and varying treatment responses make seizures a complex and often unpredictable challenge for clinicians. The management of acute seizures in children, particularly in the outpatient setting, received relatively limited attention in international treatment guidelines. Prompt and proper management in the acute setting, may prevent hospitalization as well as long-term neurological and developmental consequences.

**Methods:**

An expert consensus panel was convened to develop comprehensive and practical guidance for the early recognition, diagnostic evaluation, and management of acute convulsive seizures in children and adolescents. Recommendations were formulated through a structured review of the most recent scientific literature, current clinical guidelines, and expert opinion. Emphasis was placed on generating clear, actionable recommendations to support healthcare professionals in the rapid identification and effective treatment of acute convulsive seizures in pediatric populations.

**Results:**

This report specifically focuses on the first 5–10 min from seizure onset, which usually corresponds to the outpatient setting, including the initial steps of the inpatient treatment pathway for acute convulsive seizures in children and adolescents.

**Conclusions:**

This expert review provides actionable insights for healthcare professionals managing pediatric seizures, emphasizing the importance of a rapid and effective treatment for acute convulsive seizures.

## Background & scope

Seizures are the most common neurological disorder in children, with 4–10% of children experiencing at least one seizure by the age of 16 years. The majority of seizures occur in children under 3 years of age [1]. The wide range of causes, subtypes, and varying treatment responses make seizures a complex and often unpredictable challenge for clinicians [2]. Seizures can be either provoked or unprovoked. In this respect, a febrile seizure is a classic example of an acute provoked seizure, which does not imply the diagnosis of epilepsy. In contrast, unprovoked seizures are commonly seen in patients with epilepsy, which is a chronic disorder. In Europe, approximately 0.9 million children and adolescents are affected by active epilepsy, with a prevalence of 4.5–5.0 per 1,000 [3]. The management of acute seizures in children, particularly in the outpatient setting, has received relatively limited attention in international treatment guidelines, leaving a critical gap in treatment protocols for this vulnerable population [4]. Yet, prompt and proper management in the acute setting, may prevent hospitalization as well as long-term neurological and developmental consequences. In fact, data have shown an almost linear relationship between seizure duration and risk of secondary brain damage, underscoring the need for rapid seizure control [5]. Furthermore, recent evidence suggests that the development of easy-to-use rescue medications (i.e., buccal, intrarectal or intramuscular benzodiazepines [BZD]) dramatically reduces the number of children who need admission in an intensive care unit (ICU), as they are less likely to develop status epilepticus [6–10].

This expert review offers a comprehensive and practical guidance for early recognition, diagnosis and treatment of acute convulsive seizures in children and adolescents, with a specific emphasis on the first 5–10 min after seizure onset. This usually corresponds to the outpatient setting but may include the initial steps of the inpatient treatment pathway. These recommendations are based on the most recent literature, clinical guidelines and expert opinions, and aim to provide actionable insights for healthcare professionals managing pediatric seizures, emphasizing the importance of a rapid and effective treatment for acute convulsive seizures. A customizable seizure action plan template is provided to help physicians tailoring their interventions to the individual patient and ensuring proactive and personalized care. Evidence-based treatment insights for inpatient specialists are also included, offering comprehensive support across the different care settings.

## Methodology

These evidence-based recommendations have been developed by a panel of 9 Belgian pediatric epileptologists and are based on previous Belgian recommendations for the treatment of acute convulsive seizures in children, that have been updated and adapted to a Belgian healthcare context [11]. We conducted a comprehensive literature review using the PubMed database. The search strategy included the following terms: “acute convulsive seizures” OR “status epilepticus” AND “children” OR “pediatric”. From this extensive list of publications, we selected articles based on the following criteria: review articles or studies published in peer-reviewed journals with an impact factor ≥ 2 that specifically focused on the management of prolonged convulsive seizures or status epilepticus in the pediatric population. Application of these inclusion criteria narrowed the body of literature to approximately 100 relevant papers, which were subsequently reviewed in detail for the development of the consensus recommendations. Data from the most recent literature on this topic were complemented with expert opinions and tailored to the Belgian clinical reality. The expert panel convened on three separate occasions to review the evidence and formulate recommendations. Consensus was achieved through structured voting, with statements accepted when more than 80% of attendees expressed agreement.

## Acute convulsive seizures

An acute convulsive seizure is a sudden, involuntary event caused by abnormal, synchronized neuronal activity within brain networks. This leads to muscle jerking or stiffening, which can either impact certain body parts or the entire body, and be accompanied by loss of consciousness [12, 13]. Other common symptoms include confusion, drooling, and loss of bladder control [14]. The usual duration of a seizure ranges from a few seconds to several minutes, with different levels of severity [14]. For the caregiver, the key signs to recognize a seizure include typical muscle movements, breathing, and impaired consciousness [12]. Convulsive seizures can be provoked or unprovoked.

Febrile seizures (FS) are the typical example of provoked seizures. However, seizures may also occur in the context of an acute neurological illness (e.g., meningitis), or have a metabolic cause (e.g., hypoglycemia), requiring specific treatment in addition to antiseizure medications (ASMs). Based on the clinical presentation, FS can be simple (typical), or complex (atypical). Simple FS are the most common and occur in about 1 to 2% of the general population. Simple FS are characterized by generalized seizures, usually clonic or tonic–clonic, in infants and children between the ages of 6 months and 5 years, in the context of an increased body temperature exceeding 38ºC, often induced by a viral infection. A simple FS is defined as a seizure lasting less than 15 min, occurring only once within a 24-h period, without an associated neurological deficit, and not caused by an acute disease of the nervous system, such as encephalitis or meningoencephalitis. Importantly, simple FS per se do not cause long-term harm. In contrast, ictal febrile events characterized by focal features involving limited parts of the body and prolonged seizures are considered complex FS. Additional features of a complex FS are a duration exceeding 15 min and/or seizures occurring more than once within 24 h. Complex FS can also be associated with postictal neurological abnormalities (e.g., Todd’s paralysis) and may occur in a child with previous neurological impairment. It is estimated that 20–35% of FS can be classified as complex [15]. Of note, complex FS, especially if they are lateralized and last longer than 15 min, are often the presenting seizure type in infants with Dravet syndrome, a severe developmental and epileptic encephalopathy (DEE) characterized by fever-induced seizures and drug-resistant epilepsy associated with neurocognitive and neurobehavioral impairment. In most cases, it is caused by mutation in the *SCN1A* gene [16].

Epilepsy is a chronic disorder characterized by an enduring predisposition to generate unprovoked seizures [17]. An unprovoked seizure is a seizure that occurs without an obvious trigger, such as fever or head injury. Children can be diagnosed with epilepsy after at least two unprovoked seizures more than 24 h apart, or after a single unprovoked seizure with a high risk (≥ 60%) of recurrence within 10 years linked to specific characteristics, such as the presence of an epileptogenic brain lesion, and/or a focal seizure or epileptic discharges on electroencephalography (EEG) [2, 18]. An epilepsy diagnosis may also be established in the context of an epileptic syndrome, defined by the International League Against Epilepsy (ILAE) as a well-defined constellation of clinical features, EEG and neuroimaging findings that tend to occur together and follow a predictable course [19]. Epilepsy syndromes have a known or suspected etiology (e.g., structural, genetic, metabolic, immune, or infectious causes) and their recognition provide important information about prognosis and treatment strategies. Unlike the broader term of “epilepsy,” which focuses primarily on seizure occurrence, an epilepsy syndrome encompasses a more holistic framework, including seizure types, age of onset, comorbidities, developmental trajectory, and response to therapy, enabling a more individualized approach and enhancing diagnostic precision [20].

### Diagnostic work-up

#### Work-up after a first febrile seizure

**Fig. 1:**
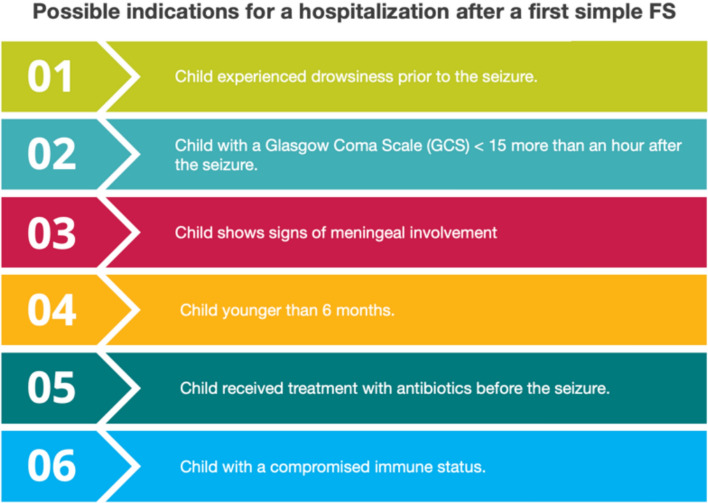
Indications for hospitalization of a child after a first simple FS

Recognizing whether a seizure is provoked or unprovoked is important to determine the appropriate work-up, treatment and prognosis. The assessment of a provoked seizure focuses on the identification of the possible trigger for the seizure. In a child with simple FS in the absence of clinical signs of encephalitis, meningitis, or septic syndrome, a lumbar puncture (LP), EEG or neuroimaging are generally not necessary [15, 21–24]. In addition, most children with simple FS do not require hospitalization. Factors that may indicate the need for hospitalization include any of these conditions: drowsiness prior to seizure, a Glasgow Coma Scale (GCS) score < 15 more than an hour after the seizure, signs of meningeal involvement, being younger than 6 months, antibiotic treatment before the FS, and a compromised immune status (Fig. 1) [25]. In contrast, the wide variability of conditions that can cause a complex FS warrants observation in the hospital, especially for patients developing such an event for the first time.

**Fig. 2:**
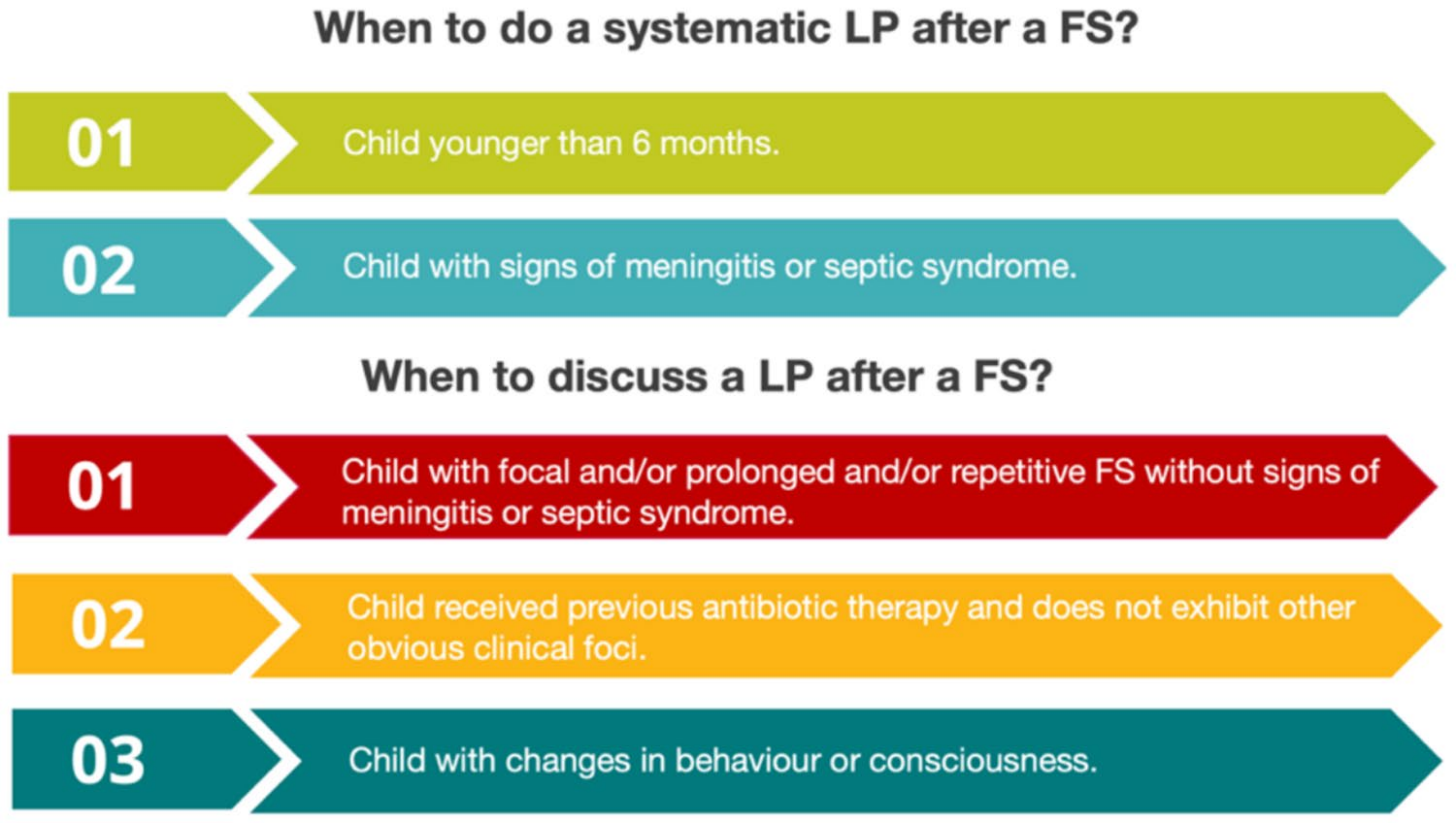
Indications for a LP in children suffering a first FS

An LP should be systematically performed in infants < 6 months and in children with signs of meningitis or septic syndrome, and it should be considered in the context of focal and/or prolonged, and/or repetitive FS, even without signs of meningitis or septic syndrome, in case of a change in behavior or consciousness (i.e. herpes encephalitis), in patients who received previous antibiotic therapy, and in the context of fever of unknown origin (Fig. 2).

Importantly, a previous neurodevelopmental abnormality, or a family history of FS or epilepsy should not play a role in the decision whether or not to perform an LP. An LP is contraindicated when a space-occupying intracranial lesion is suspected because of the risk of brain herniation, as well as in cases of hemodynamic instability, significant coagulation disorders, or severe thrombocytopenia. There is no consensus regarding the utility of an early EEG in the context of complex FS. In fact, while some studies suggest that an abnormal EEG with epileptiform abnormalities has a low predictive value for subsequent epilepsy, others have reported the opposite [26, 27]. EEG can help in diagnosing herpes simplex encephalitis, the most common form of sporadic encephalitis worldwide, by showing diffuse or focal slowing and/or distinctive high-voltage, 1-cycle-per-2-to-3 s lateralized periodic discharges (LPDs) [24, 28, 29]. A Brain MRI is highly recommended if there is a suspicion of a congenital brain malformation and/or herpes encephalitis. If there is a suspicion of a space-occupying lesion or herniation and a brain MRI cannot be performed, a head CT scan can be performed in the emergency setting. However, the likelihood of identifying a lesion on neuroimaging that requires immediate neurosurgical or medical intervention is extremely low, making this investigation unnecessary for most children with complex FS [30].

#### Initial work-up after a first unprovoked seizure

For an unprovoked seizure, the clinical assessment should include a detailed seizure description, family history for seizures and neurological disorders, and a neurological examination. Standard diagnostic evaluation in children typically includes an EEG recording including both wakefulness and sleep states, with a minimum duration of 20 min of wakefulness, without significant artefacts, and 30 min of sleep [31]. Brain imaging is recommended in the emergency setting following a first unprovoked seizure to guide appropriate acute management, particularly in patients presenting with abnormal neurological findings, or focal seizure onset. In those cases, there is a risk of underlying structural abnormalities (e.g., hemorrhage, mass lesion) that may require urgent intervention. While non-contrast CT scan can rapidly detect certain pathologies, such as large tumors, stroke, calcifications, or arteriovenous malformations, it does involve exposure to ionizing radiation, which is a notable concern, especially in a pediatric population. Conversely, brain MRI, which does not involve radiation, offers superior soft tissue resolution and is the preferred imaging modality. Contrast-enhanced CT may be considered if infection or small neoplasms are suspected and MRI is not immediately accessible.

### Duration and characteristics of the post-ictal state

The post-ictal state refers to the period starting right after a seizure subsides and ending when the patient returns to baseline. It typically lasts between 5 and 30 min and is characterized by an altered mental status including symptoms such as disorientation, confusion, drowsiness, headache and nausea [32–35]. Post-ictal deficits in children with focal seizures with impaired consciousness may take 1 to 2 h to resolve. However, Todd’s paralysis, that is a temporary weakness or paralysis affecting the part of the body involved in the seizure, may take up to 2 days to fully resolve. Determining the end of the seizure and the beginning of the post-ictal state is typically easy in case of acute convulsive seizures. With respect to the localization, a post-ictal hemiparesis points towards a contralateral seizure onset, whereas postictal aphasia indicates involvement of the dominant hemisphere. Patients presenting with a new focal sign (e.g., unilateral weakness) after an unwitnessed but suspected seizure may require brain imaging to rule out a lesion [36].

Nonconvulsive status epilepticus should always be considered in patients with prolonged postictal confusion [36]. An EEG can help to distinguish between these two states [37]. A post-ictal EEG is typically characterized by attenuation or slowing (usually in the delta frequency range), or a combination of both [36]. As further recovery ensues, the delta slowing transitions to theta frequencies before a normal background rhythms returns. Some patients fall asleep during their recovery. The location of the post-ictal slowing/attenuation may help in the identification of the region involved by the seizure. Changes in the EEG tend to be more pronounced with prolonged seizures. However, in these cases an EEG has less lateralizing value [36]. A retrospective study demonstrated that the average time for an EEG to return to baseline after a seizure was 120 min, with a maximum of 300 min in children [38]. In contrast, an EEG in nonconvulsive status epilepticus typically shows continuous or near-continuous epileptiform discharges (e.g., spikes, sharp waves, or rhythmic spike-and-wave activity) lasting for minutes to hours without returning to baseline [39]. The experts conclude that if a child remains unresponsive or does not regain consciousness within 30 min to 1 h, it should be considered a warning sign and an EEG should be performed to identify and eventually treat ongoing seizures.

### Assessing the need and urgency of care: exceptional circumstances requiring an urgent medical intervention

**Fig. 3:**
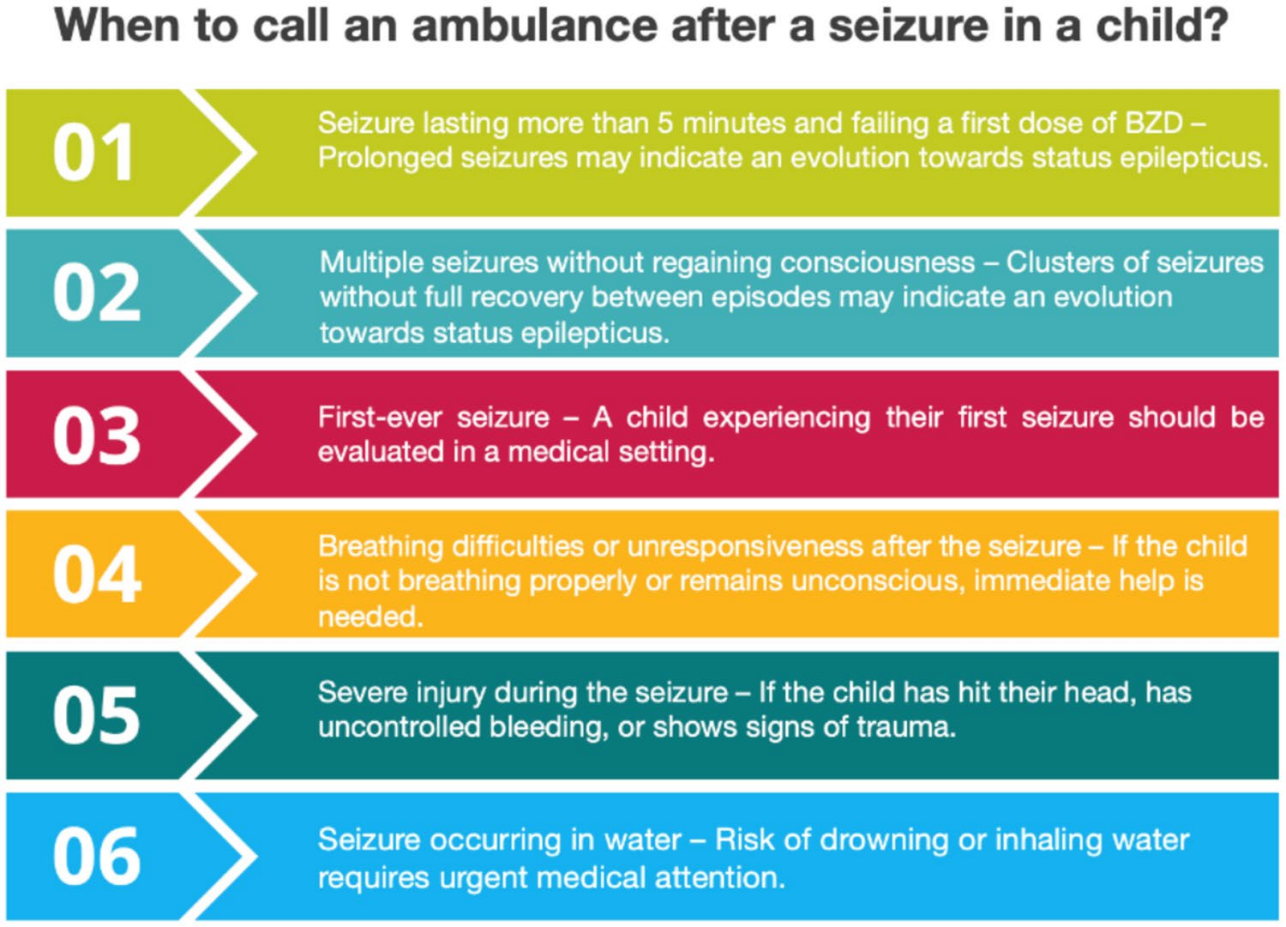
Situations requiring an urgent medical intervention after a seizure

There are no comprehensive guidelines that specify the exact circumstances and timing for an urgent medical intervention during a seizure. Therefore, the panel agreed that this decision should always be made on a case-by-case basis. An overview of situations in which emergency medical services should be contacted is shown in Fig. 3. Important factors to take into consideration in this decision are the seizure history and/or epilepsy type of the child. As demonstrated by *Shinnar *et al*.*, the duration of an initial seizure in children strongly predicts the length of subsequent seizures [5, 40].

## Treatment of seizures

The management of acute convulsive seizures in children requires a structured, timeline-based approach. As for any medical emergency, the first step should be an assessment of Airway, Breathing, and Circulation (ABC) [12, 41]. Ensuring that the airway is clear, checking for proper breathing, monitoring circulation and ensuring that the child is in a side position are critical to the child’s safety. In addition to these priorities, timing the seizure duration, and staying with the child until they fully recovers are consistent actions throughout the process [41].

**Fig. 4:**
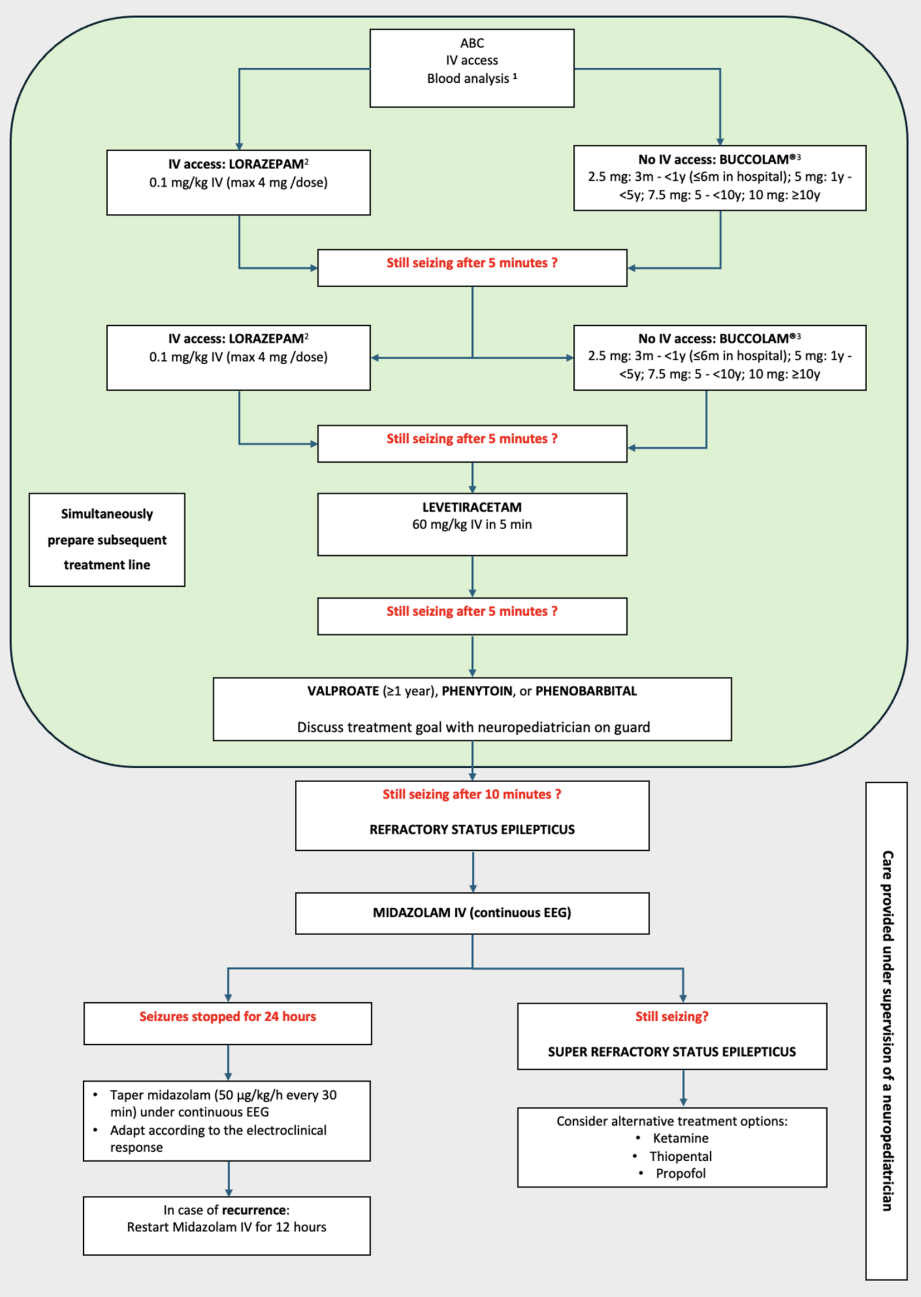
Consolidated treatment flow for patients with acute convulsive seizures. 1. Blood test and pH: complete blood count, dose of antiepileptic drugs, glycemia, assess ion levels (Na, K, Cl, HCO3-, Ca, Mg), ALT level, coagulation, blood culture if fever. 2. When lorazepam is not available or feasible: Midazolam IV (0.1 mg/kg, max 10 mg/dose) or Diazepam IV (0.2 mg/kg, max 10 mg/dose). 3. When Buccolam.® not available or feasible: Midazolam intranasal (0.3 mg/kg, max 5 mg/nostril) or intramuscular (0.2 mg/kg, max 10 mg/dose) or Diazepam intrarectal (0.5 mg/kg, max 20 mg/dose).

Treatment decisions depend on seizure duration. Status epilepticus is operationally defined as seizures lasting more than 5 min, while the epidemiological definition uses a duration of 30 min. [42, 43] It has been shown that irreversible cerebral metabolic consequences may occur after 30 min of ongoing seizures [44]. On the other hand, most seizures are often short and self-limiting, resolving spontaneously within 5 min from onset, making the use of rescue medications, typically benzodiazepine (BZD), unnecessary. An exception is represented by children suffering from either frequent, brief seizures with preserved consciousness between episodes, children who are known to suffer from prolonged seizures, or children with severe epilepsy syndromes prone to recurrent refractory status epilepticus (e.g., Dravet syndrome). In those patients, the administration of a rescue BZD at onset, without further waiting, is strongly suggested [12]. Some experts argue that the administration of a rescue BZD can already be considered after 2 min of ongoing seizures, particularly if convulsive. This may reduce the stress experienced by the family and the psychosocial trauma of witnessing their child seizing without intervening. If a seizure persists beyond 5 min (i.e., early status epilepticus according to the ILAE) a BZD should be administered promptly by parents or a caregivers [12, 40, 42, 45–47]. If the seizure does not stop within 3 to 5 min after the first administration of an adequate dose of BZD, an ambulance should be called, as it is not recommended to administer additional doses of BZD without medical or paramedical supervision, due to the risk of respiratory depression [46]. If the seizure continues, second- and third-line treatments may be necessary. Below, we present contemporary recommendations following the chronological steps in the treatment plan (See Fig. 4 for a consolidated, schematic overview).

### First-line treatment: benzodiazepines

As previously explained, if a seizure lasts longer than 5 min, [12] an adequate dose of BZD should be administered (Tables 1 & 2). When administered early during the seizure (i.e., within 5 min from onset), BZDs have shown to be effective in controlling seizures in about 80% of cases [48]. The choice of the BZD should be made based on its ease of administration, effectiveness, and safety.

**Table 1:** Availability of first-line medications for acute convulsive seizures in Belgium

	Administration route	Marketed in belgium (Yes/No)	In-label / Off-label Use	Additional information
*Midazolam*	IB	Yes	In-label	Indicated for prolonged, acute convulsive seizures: 3 m—< 18 y Note: Children of 3–6 months must be treated in a hospital with monitoring and resuscitation equipment
*Midazolam*	IV/IM/IO	Yes	Off label	N.A
*Midazolam*	IN	No	N.A	N.A
*Lorazepam*	SL	Yes	Off label	Off label for both indications
*Lorazepam*	IV/IM	Yes	Off label	N.A
*Diazepam*	IV/IR	Yes	In-label	Indicated for: - Status epilepticus: 0-18y - Febrile seizures: 0-18y
IB: intrabuccal, IV: intravenous, IM: intramuscular, IN: intranasal, SL: Sublingual, IR: intrarectal, IO: intraosseous, m: months, y: years

**Table 2:** Dosing of first-line medications for acute convulsive seizures

	Route	Dose
*Midazolam*	IB	6 months – < 1 year: 2.5 mg 1 – < 5 years: 5 mg 5 – < 10 years: 7.5 mg ≥ 10 years: 10 mg
	IM/IV/IO	0.1—0.2 mg/kg (max single dose: 10 mg)
	IN	0.2—0.3 mg/kg (max single dose: 10 mg)
*Lorazepam*	IV/IO	0.1 mg/kg (max single dose: 4 mg)
	IN	0.1 mg/kg
*Diazepam*	IV/IO	0.1—0.3 mg/kg (max single dose: 10 mg)
	IR	2 – 6 years: 0.5 mg/kg 6 – 12 years: 0.3 mg/kg > 12 years: 0.2 mg/kg

Recently, buccal midazolam has become the preferred option, largely replacing the use of intrarectal diazepam, due to its ease of administration both in and outpatient settings and its favorable safety profile [46, 49]. While sublingual lorazepam tablets were used in the past, they are no longer recommended [30, 41, 46, 50–52]. In fact, sublingual lorazepam has a slower absorption than buccal midazolam. In addition, its use in children is off-label (*see *Table 1) [53, 54]. In the inpatient context when an intravenous (IV) line is present, lorazepam or midazolam should be administered intravenously [46]. Lorazepam has a lower lipophilicity than midazolam, allowing it to last longer in the central nervous system (CNS) (i.e. 4 h *vs*. 30 min for midazolam) [55]. The slower clearance of lorazepam out of the brain results in a longer duration of the therapeutic effect. This longer treatment effect theoretically reduces the risk for seizure recurrence during the critical early phase after administration and may reduce the need for repeated doses. [55].

Other potential routes of administration for BZD are intrarectal (IR), intranasal (IN), intramuscular (IM) or intraosseous (IO) (Table 1). However, these administration routes should only be considered when buccal or IV administration is not possible. Compared to buccal midazolam, rectal diazepam is less effective and more cumbersome to administer. Furthermore, although it has historically been considered a simple treatment option that could be widely used in outpatient community settings, the rectal route has a low level of acceptance, particularly among older children. [6, 50] However, when only rectal formulations are available, or for patients in whom a buccal administration is not feasible, rectal diazepam remains a viable option [46]. The IN route may provide an effective alternative for buccal formulations in patients with issues, such as jaw clenching, hypersalivation, or uncontrollable swallowing during their seizures [50, 51]. IM administration has a slower onset of action, yet it can serve as a practical and effective alternative for IV administration [56]. However, administering IM injections can be challenging and should therefore only be performed by a medically trained person. [52]. For children who do not stop seizing within 5 min after the first dose of BZD, a second dose of BZD should be administered. Importantly, due to the risk of respiratory depression, especially in younger children, we recommend to perform this second administration in the presence of a healthcare professional (HCP). In selected patients, mainly children who suffer from recurrent clusters of seizures, and after discussion with the treating physician, the second BZD may also be given by a parent or caregiver.

### Second-line treatment: what to do in case of BZD failure

If the seizure continues after two BZD administrations, a second-line treatment with levetiracetam, valproate, phenobarbital, lacosamide or phenytoin is recommended. The administration of this second-line treatment should always be done by a physician.

Levetiracetam is often favored as a second-line treatment due to its ease of preparation, simple administration, speed of infusion (i.e., 5 min) and generally favorable safety profile [46, 57, 58]. Randomized controlled trials (RCTs) and meta-analyses indicate that the efficacy of levetiracetam is comparable to that of other treatments [46, 52, 57, 59–64]. For instance, the ESETT trial showed similar efficacy and safety of levetiracetam, fosphenytoin, and valproate in status epilepticus [61, 65]. Two RCTs and a retrospective study found levetiracetam to be even superior to phenytoin [66–68]. The use of valproate comes with specific potential risks, including liver failure in mitochondrial disorders that may be still undiagnosed when the status epilepticus occurs. Additionally, caution is warranted when valproate is used in children with a liver disease [51]. While many protocols still mention an infusion time of 20 min for valproate, evidence suggests that it is safe and tolerable to administer this agent as a rapid infusion in 5 min. [52].

Phenytoin is another ASM that can be used as second-line treatment for status epilepticus. While historically phenytoin has been considered a standard first- or second-line treatment for status epilepticus, its use has declined in recent years due to the risk of acute cardiac hemodynamic changes, particularly with rapid IV infusion.

Similar to phenytoin, phenobarbital has been used as a first- and second-line treatment for over 50 years [69, 70]. It is well tolerated and offers rapid, long-lasting effects with a favorable safety profile, even when used at higher doses. However, given the paucity of evidence from RCTs, current guidelines provide weaker recommendations for its use.

Lacosamide, is infrequently used as a second-line option for the treatment of acute convulsive seizures in children. However, a recent study suggest that it may represent an alternative to levetiracetam or valproate in this setting [71]. Further research is required to confirm these findings and better delineate the potential place of lacosamide in the treatment of pediatric status epilepticus [72, 73]. Regarding doses, literature provides a range of recommendations for each medication, as described in Table 3. [57, 65, 74, 75]

**Table 3:** Dosing of second-line treatment of antiseizure medications

	Dose range (mg/kg)	Max. dose (mg)	Infusion speed (min)
*Levetiracetam*	60*	4500	5
*Valproate*	40	3000	5
*(Fos)phenytoin*	15–20	1500–2000	20* under ECG monitoring and in saline solution
*Lacosamide*	2–4 or 6–8	Not specified	20
*Phenobarbital*	15–20	Not specified	15

*Levetiracetam: New evidence suggests that 60 mg/kg is the most effective dose.^*(63, 64, 76)*^

### Third-line treatment & intensive care options

When also the second-line treatments fail, patients enter the refractory phase of status epilepticus. For these severe cases admitted in the ICU, third-line treatment may consist of general anesthesia. It is highly recommended to monitor the effects of anesthetics on the brain with continuous EEG, providing guidance for the required depth of pharmacologically induced coma to control seizures [46, 48].

The preferred treatment in this setting consists of IV midazolam [77]. This agent is generally preferred over other IV BZDs, including lorazepam, given its shorter half-life and its higher lipophilicity. These properties allow for rapid redistribution out of the brain with a relatively short duration of action on the brain. In case of refractory status epilepticus (RSE), medications with a short half-life in the CNS are ideal for continuous infusion, as they allow for precise titration and effective neurological monitoring [55]. A recent pediatric study emphasizes that low-dose midazolam continuous infusion (< 0.2 mg/kg/h) minimizes the risk of respiratory depression, avoiding the need for ICU admission and intubation [77]. Apart from high-dose BZDs, other types of anesthetics can also be considered in the ICU, including isoflurane, ketamine and barbiturates (thiopental, pentobarbital). To date, however, there are no RCT data that can steer the choice for a specific anesthetic drug in patients with RSE, resulting in varying dosing protocols (Table 4) [78–80].

**Table 4:** Bolus doses and continuous infusion rates for the treatment of refractory status epilepticus

	Bolus dose (mg/kg)	Continuous infusion (mg/kg/h)
*Midazolam*	0.2–0.5	0.1–0.3*
*Thiopental*	2–7	0.5–5
*Propofol*	1–2	0.3–12
*Ketamine*	0.5–3	0.1–10
*Pentobarbital*	5–15	0.5–5

* IV 0.2 mg/kg bolus, 0.1 mg/kg/h IV thereafter (max 1.8 mg/kg/h). Titrate as needed by 0.1 mg/kg/h every 5 min until convulsive status epilepticus is controlled (0.2 mg/kg bolus at every increase)

More recently, combinations of anesthetics such as propofol-ketamine or midazolam-ketamine as third-line therapy has shown positive results in RSE, (Fig. 4). as their early initiation may reduce side effects linked to high doses of propofol or barbiturates [81–83].

## Conclusions

**Fig. 5:**
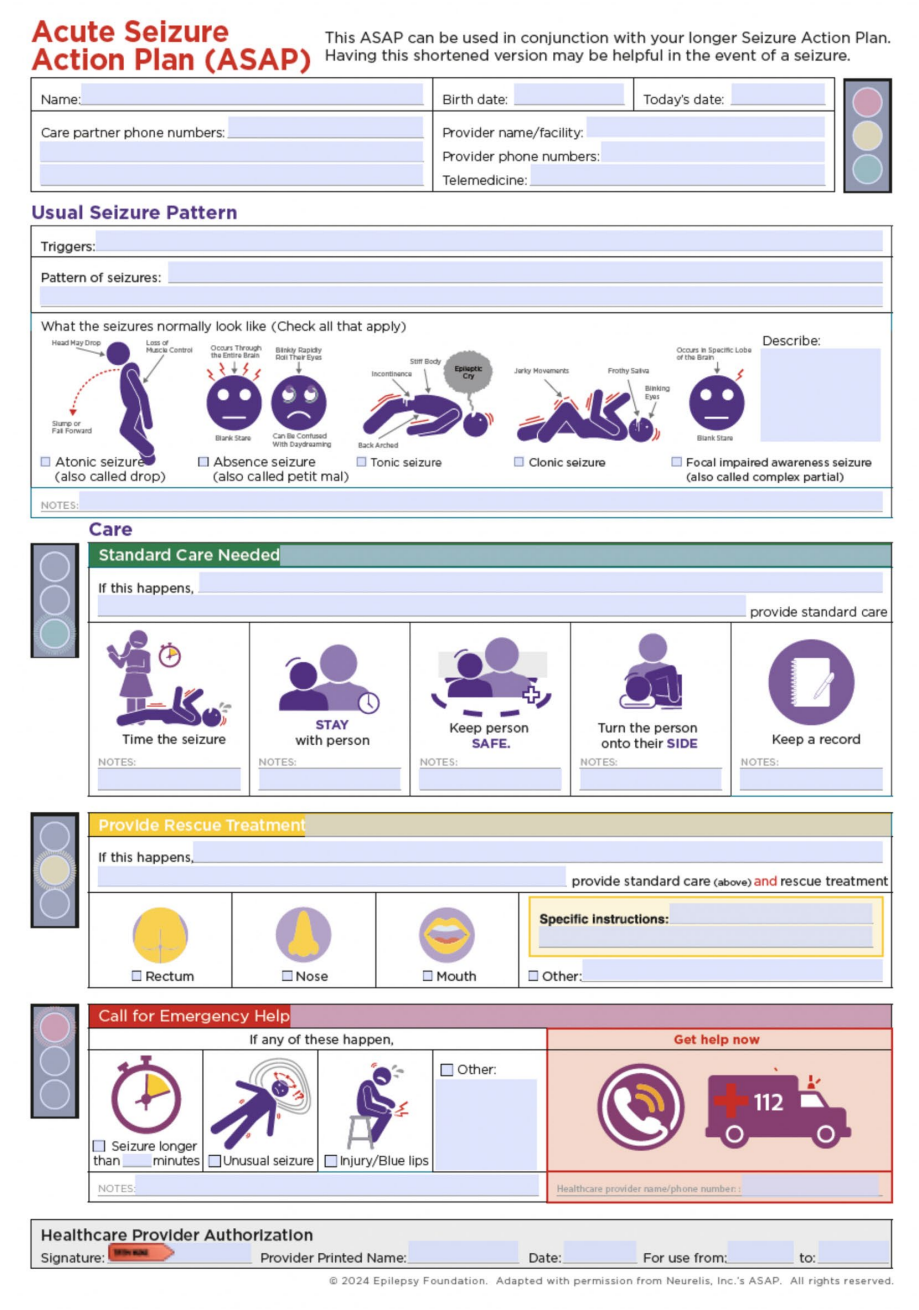
This template is adapted from resources developed by the Epilepsy Foundation (United States), with acknowledgment of the original source

For an effective long-term seizure management, it is critical to secure proactive measures to address potential recurrences, creating the need for customized action plans in seizure management. Providing personalized seizure action plans empowers caregivers and facilitates timely interventions when seizures occur. This is especially recommended for patients with a history of convulsive seizures of > 5 min. [5, 84] The panel unanimously agreed on the need to provide each patient with their own personalized seizure action plan, even for FS, based on their seizure history and typical seizure duration. To support this practice, we propose a standardized template for acute seizure action plans (Fig. 5).

## Data Availability

No datasets were generated or analysed during the current study.
